# Iron Deficiency and Sleep/Wake Behaviors: A Scoping Review of Clinical Practice Guidelines—How to Overcome the Current Conundrum?

**DOI:** 10.3390/nu16152559

**Published:** 2024-08-03

**Authors:** Scout McWilliams, Olivia Hill, Osman S. Ipsiroglu, Stefan Clemens, Alexander Mark Weber, Michael Chen, James Connor, Barbara T. Felt, Mauro Manconi, Andre Mattman, Rosalia Silvestri, Narong Simakajornboon, Susan M. Smith, Sylvia Stockler

**Affiliations:** 1 H-Behaviours Research Lab (Previously Sleep/Wake-Behaviours Research Lab), BC Children’s Hospital Research Institute, Department of Pediatrics, University of British Columbia, Vancouver, BC V5Z 4H4, Canada; scout.mcwilliams@bcchr.ca (S.M.); olivia.hill@bcchr.ca (O.H.); sstockler@cw.bc.ca (S.S.); 2 Divisions of Developmental Pediatrics, Child and Adolescent Psychiatry and Respirology, BC Children’s Hospital, Department of Pediatrics, University of British Columbia, Vancouver, BC V6T 1Z4, Canada; 3 Department of Physiology, Brody School of Medicine, East Carolina University, Greenville, NC 27834, USA; clemenss@ecu.edu; 4 Department of Pediatrics, University of British Columbia, Vancouver, BC V6T 1Z4, Canada; aweber@bcchr.ca; 5 BC Children’s Hospital Research Institute, Vancouver, BC V5Z 4H4, Canada; 6 Department of Pathology and Laboratory Medicine, University of British Columbia, Vancouver, BC V6T 1Z4, Canada; michael.chen@islandhealth.ca (M.C.); amattman@providencehealth.bc.ca (A.M.); 7 Department of Neurosurgery, Penn State Hershey Medical Center, Hershey, PA 17033, USA; jconnor@pennstatehealth.psu.edu; 8 Department of Pediatrics, University of Michigan, Ann Arbor, MI 48109, USA; truefelt@med.umich.edu; 9 Sleep Medicine Unit, Neurocenter of the Southern Switzerland, Regional Hospital of Lugano, Faculty of Biomedical Sciences, Università della Svizzera Italiana, 6900 Lugano, Switzerland; mauro.manconi@eoc.ch; 10 Department of Neurology, University of Bern, 3012 Bern, Switzerland; 11 Department of Clinical and Experimental Medicine, Sleep Medicine Center, University of Messina, Azienda Ospedaliera Universitaria “Gaetano Martino”, 98122 Messina, Italy; rosalia.silvestri@unime.it; 12 Sleep Center, Cincinnati Children’s Hospital Medical Center, Cincinnati, OH 45229, USA; narong.simakajornboon@cchmc.org; 13 Department of Nutrition, UNC-Nutrition Research Institute, University of North Carolina at Chapel Hill, Kannapolis, NC 28081, USA; susan_smith@unc.edu; 14 Division of Biochemical Diseases, Department of Pediatrics, BC Children’s Hospital, University of British Columbia, Vancouver, BC V6T 1Z4, Canada

**Keywords:** attention deficit hyperactivity disorder, brain iron, central iron deficiency, clinical practice guidelines, iron deficiency, restlessness, restless legs syndrome, sleep disorders

## Abstract

Current evidence suggests that iron deficiency (ID) plays a key role in the pathogenesis of conditions presenting with restlessness such as attention deficit hyperactivity disorder (ADHD) and restless legs syndrome (RLS). In clinical practice, ID and iron supplementation are not routinely considered in the diagnostic work-up and/or as a treatment option in such conditions. Therefore, we conducted a scoping literature review of ID guidelines. Of the 58 guidelines included, only 9 included RLS, and 3 included ADHD. Ferritin was the most frequently cited biomarker, though cutoff values varied between guidelines and depending on additional factors such as age, sex, and comorbidities. Recommendations surrounding measurable iron biomarkers and cutoff values varied between guidelines; moreover, despite capturing the role of inflammation as a concept, most guidelines often did not include recommendations for how to assess this. This lack of harmonization on the interpretation of iron and inflammation biomarkers raises questions about the applicability of current guidelines in clinical practice. Further, the majority of ID guidelines in this review did not include the ID-associated disorders, ADHD and RLS. As ID can be associated with altered movement patterns, a novel consensus is needed for investigating and interpreting iron status in the context of different clinical phenotypes.

## 1. Introduction

**Figure 1:**
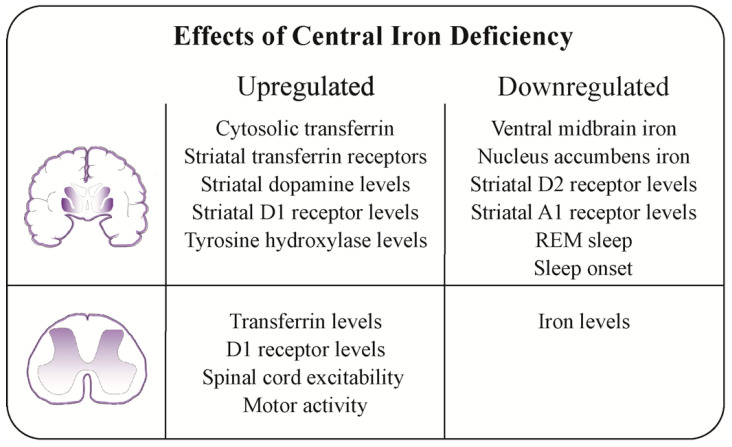
Effects of central iron deficiency (CID), as grouped by region of interest, brain, and spinal cord. CID refers to low iron levels in both the brain and spinal cord. Highlighted here are areas in the brain and spinal cord that are particularly susceptible to CID-induced changes in dopamine modulation. In the striatum, they are suspected to modulate motor outputs, while in the dorsal spinal cord, they are associated with sensory inputs. Data compiled from Silvani et al. [2] and Woods et al. [3]. A1 receptor: adenosine receptor; D1/D2 receptor: dopamine receptors; REM: rapid eye movement.

Iron deficiency (ID) is the most common micronutrient deficiency in the world and one of the biggest contributors to the global prevalence of anemia. Children, females of reproductive age, and those living in low-income countries or of low socioeconomic status are disproportionately affected by ID [1]. Besides its essential roles in hemoglobin formation and hematopoiesis, energy generation, inflammation, remission, and healing, iron has numerous functions within the central nervous system, for example, as a cofactor for various enzymes, particularly within the dopaminergic and glutamatergic systems. The effects of central ID (CID) on measurable neurotransmitters, receptor levels, excitability, and behaviors have been illustrated in Figure 1 [2,3]. Further, animal studies have proven the importance of iron in brain development and functioning [4]. In humans, maternal ID in pregnancy, particularly in the first trimester, has been associated with low birth weight and preterm delivery, as well as an increased risk of intellectual disability, autism spectrum disorder (ASD), and attention deficit hyperactivity disorder (ADHD) in the offspring [5,6].

Importantly, ID can occur in the absence of anemia and is referred to as non-anemic iron deficiency (NAID). While much of the focus has historically been on ID anemia (IDA), recent evidence has shown that NAID can have significant consequences on health and wellbeing. NAID has been associated with neurodevelopmental disorders such as ADHD and a number of sleep disorders, such as restless legs syndrome (RLS), periodic limb movements in sleep (PLMS), and restless sleep disorder (RSD), all of which may profit from iron supplementation as a therapeutic intervention [7,8,9,10,11]. These disorders typically present with restlessness in sleep and wake and during transitions from wake to sleep states. While the specific mechanisms by which NAID contributes to symptoms in the aforementioned conditions are not fully understood, current evidence in humans by imaging studies suggests that CID may be an important factor (Table 1), which may not be reflected by peripheral iron levels.

Rodent models utilizing diet modifications show the causal links between ID and alterations in dopamine transmission that are consistent with a hyperdopaminergic hypothesis of RLS [2]. Despite this strong evidence of possible biological interactions, there is a general paucity of ADHD animal models that incorporate ID or findings from RLS models. Intriguingly, data from the spontaneously hypertensive rat model present the preclinical characteristics of both RLS and ADHD [12]. As hypertension is regulated in part by the autonomic nervous system and iron levels [13], this may indicate an additional layer of complexity in the regulation of ADHD and RLS and further justifies the terminology of CID syndromes to facilitate more in-depth clinical phenotyping of restlessness, as well as blood work investigations for the treatment of sleep disorders such as RLS, PLMS, and RSD and also ADHD [7,10,14]. The association between iron homeostasis and restlessness in sleep and wake states raises questions about which iron biomarkers should be measured, the timing of such measurements, and how the results should be interpreted.

**Table 1:** Summary of MRI brain iron studies in ADHD and RLS. ADHD: attention deficit hyperactivity disorder; MFC: magnetic field correlation; mo: months; PLMS: periodic limb movements of sleep; QSM: quantitative susceptibility mapping; RLS: restless legs syndrome; ROI: regions of interest; SF: serum ferritin, Y: years.

Author	Study Population			Method	Results
	Condition	Participant Number	Age (Y): Range Mean		
Adisetiyo et al., 2014 [15]	ADHD	*n* = 22 ADHD *n* = 27 controls	8–18 12.7 13.3	MRI imaging relaxation rates (R2, R2*, R2′) and magnetic field correlation (MFC) in the globus pallidus, putamen, caudate nucleus, and thalamus -R2, R2*, R2′ -MFC	-No difference in R values -Lower MFC in ADHD (lower brain iron)
Adisetiyo et al., 2019 [16]	ADHD	*n* = 30 ADHD *n* = 29 controls	8–18 14.0 13.9	-R2* -MFC	-No difference in R2* or MFC -Increased R2* and MFC (increased brain iron) with psychostimulant use duration in ADHD more than with age
Allen et al., 2001 [17]	RLS	*n* = 5 RLS *n* = 5 controls	66.2 66.4	-R2′	-Lower R2′(lower brain iron) in RLS and in proportion to RLS severity
Astrakas et al., 2008 [18]	RLS	*n* = 25 RLS *n* = 12 controls	55–82 66.5 54–89 65.7	-T2	-Higher T2 (lower brain iron) in RLS
Beliveau et al., 2022 [19]	RLS	*n* = 72 RLS *n* = 72 controls	46–59 51.9 (median) 51.0 (median)	-R2, R2′, and R2* -QSM	-Higher R and QSM values (increased iron brain iron) in RLS
Cortese et al., 2012 [20]	ADHD	*n* = 18 ADHD *n* = 9 controls *n* = 9 psychiatric controls	118.8 mo 120.8 mo 123.5 mo	-T2*	-Higher T2* (lower brain iron) in ADHD -SF and T2* values did not correlate significantly in most regions
Earley et al., 2006 [21]	RLS	*n* = 22 early-onset RLS *n* = 19 late-onset RLS *n* = 39 controls	57.1 67.4 60.5	-R2′	-Lower R2′ (lower brain iron) in early-onset RLS symptoms, but not late-onset RLS
Godau et al., 2008 [22]	RLS	*n* = 6 RLS *n* = 19 controls	47–68 60 59	-T2	-Higher T2 (lower brain iron) in RLS
Hasaneen et al., 2017 [23]	ADHD	*n* = 17 ADHD *n* = 18 controls	6–15 8.4 8.5	-R2*	-Lower R2* (lower brain iron) in ADHD which correlated with ADHD type but not with ADHD severity
Knake et al., 2010 [24]	RLS	*n* = 12 RLS *n* = 12 controls	43–46 58.5 41–74 56.8	-T2	-No difference in T2 values
Li et al., 2016 [25]	RLS	*n* = 39 RLS *n* = 29 controls	58.4 57.9	-QSM	-Lower magnetic susceptibility (lower brain iron) in RLS and possible connection to PLMS
Margariti et al., 2012 [26]	RLS	*n* = 11 RLS *n* = 11 controls	48–70 55.3 42–73 56.1	-T2	-Lower T2 (higher brain iron) in RLS
Moon et al., 2014 [27]	RLS	*n* = 37 RLS *n* = 20 early-onset RLS *n* = 17 late-onset RLS *n* = 40 RLS controls *n* = 20 early-onset controls *n* = 20 late-onset controls	50.3 58.1 47.0 59.4	-T2	-Higher T2 (lower brain iron) in late-onset RLS, but not early-onset RLS
Moon et al., 2015 [28]	RLS	*n* = 37 RLS *n* = 40 controls	53.8 53.2	-R2, R2*, and R2′	-Relaxometry and ROI determination methods significantly influenced the outcome of brain iron estimates
Rizzo et al., 2013 [29]	RLS	*n* = 15 RLS *n* = 15 controls	51.0 51.0	-Phase from gradient-echo scan	-Higher phase values (lower brain iron) in RLS

Historically, ID was diagnosed solely based on hematologic markers such as hemoglobin in conjunction with, for example, mean corpuscular volume and mean corpuscular hemoglobin. Over the last two decades, however, iron-specific markers, most notably serum ferritin (SF), have become the mainstay for diagnosing NAID and IDA. Since 2001, the World Health Organization (WHO) has recommended SF as the primary biomarker for the assessment of iron status [30]. SF is an iron storage protein and acute phase reactant that is present in all cells. While low SF concentrations indicate low iron stores, normal or elevated SF does not exclude ID in cases of infection or inflammation [1].

We carried out a scoping literature review with the aim to investigate clinical ID guidelines, specifically looking at recommendations for diagnosis, such as iron biomarkers, their cutoff values, and the role of inflammation when interpreting laboratory results. Our secondary aim was to identify whether common disorders presenting with restlessness such as ADHD and RLS have been incorporated into the clinical guidelines, as diagnosis and treatment can have significant implications for the affected individuals. A scoping review was the agreed-upon methodology given the exploratory nature of this project and to facilitate the identification of gaps in clinical knowledge.

## 2. Materials and Methods

This scoping review was carried out in accordance with the PRISMA-Scr Checklist (Appendix A, Table A1). A protocol for this review does not exist. Two reviewers (SM and OH) were involved in identifying guidelines for inclusion. The initial search was carried out on 27 June 2020 in CINAHL, Embase, and Medline with no date restrictions and was updated on 7 April 2023. The search strategy included variations of the terms “iron deficiency” and “guideline”; the detailed search strategy is laid out in Table 2. Covidence, a web-based collaboration software platform that streamlines the production of systematic and other literature reviews (https://www.covidence.org/, accessed on 18 May 2024), was employed for the selection and de-duplication process. The search was updated in 2023. Additional guidelines were identified by searching Trip Medical Database (https://www.tripdatabase.com/, accessed on 18 May 2024) as well as conducting Google searches and checking websites of medical organizations. Two reviewers (SM and OH) carried out data extraction and organized data into a spreadsheet. A third reviewer (OI) was available to oversee this process.

### 2.1. Inclusion Criteria

Guidelines were included if (1) they were general ID guidelines, defined as those which targeted a general population (e.g., adults, elderly, children, ethnic groups), pregnancy-specific ID guidelines, and disease-specific guidelines (e.g., chronic kidney disease (CKD)); (2) the guideline or consensus paper was created by/on behalf of a larger governing body (e.g., international, national, or regional organizations/societies); (3) they were available in English-language.

### 2.2. Exclusion Criteria

Guidelines were excluded if they were (1) opinion papers or guidelines published by authors not affiliated with a larger governing body; (2) reviews of clinical guidelines.

### 2.3. Data Analysis

Three reviewers (SM, OH, and OI) were involved in analyzing the extracted data. A qualitative analysis was carried out by reviewing the following categories:

1 Population defined by age, pregnancy status, and medical conditions. Guidelines were organized into three categories: (1) general ID, (2) ID in pregnancy, and (3) disease-specific ID.
  General ID guidelines were defined as those guidelines which could be applied to a general population and which may have included specific subpopulations within the guideline.
  Disease-specific ID guidelines were defined as those guidelines which dealt with only a specific population, namely chronic disease populations, in which the diagnosis and management of ID is different from general ID guidelines.
1.1 Year and country of publication.
2 Associated clinical presentations, conditions, diagnoses, and risk factors for ID.
2.1 If ADHD and/or RLS were included as either signs/symptoms or as being associated with ID. Guidelines that used broad terminology such as “behavioral disturbances” or “sleep disturbances” without specifying the aforementioned conditions were not classified as having included ADHD and/or RLS.
3. Suggested cutoff values for SF, taking into account age- and sex-specific cutoff values.
3.1 Additional iron and hematologic biomarkers included in the guidelines. Examples of iron biomarkers (other than SF) are serum iron and transferrin saturation, while hematologic biomarkers include hemoglobin and mean corpuscular volume/mean corpuscular hemoglobin.

## 3. Results

**Figure 2:**
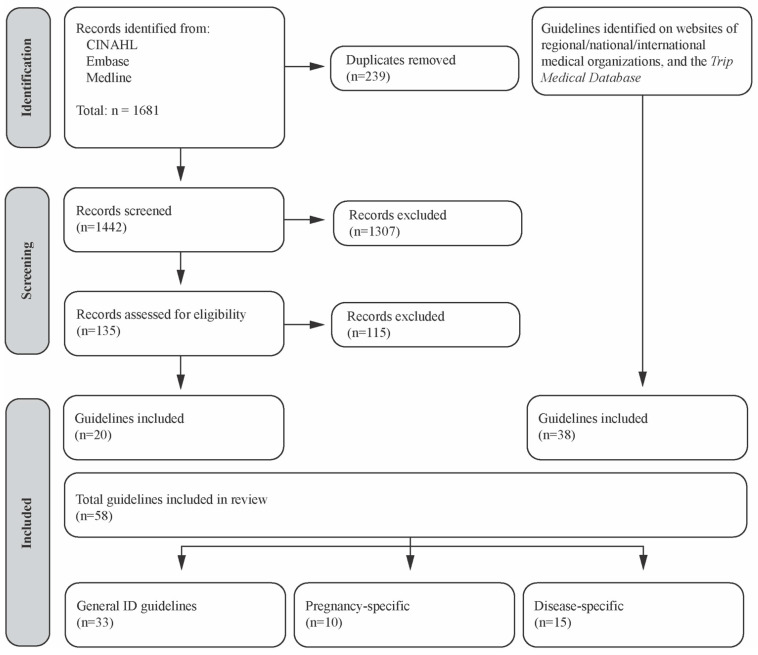
Study selection. Adapted from [88].

A total of 58 ID guidelines were identified; 20 identified through the electronic databases, 36 identified through searching websites of international, national, and regional health organizations, and 2 identified on Trip Medical Database. A total of 33 were general ID guidelines, 10 were pregnancy-specific, and 15 were disease-specific (Figure 2; Table A2, Table A3 and Table A4). The included guidelines were published between 1989 and 2023 with the majority published in the 2010s (*n* = 36) [31,32,33,34,35,36,37,38,39,40,41,42,43,44,45,46,47,48,49,50,51,52,53,54,55,56,57,58,59,60,61,62,63,64,65,66]. Thirteen were published in the 2020s [67,68,69,70,71,72,73,74,75,76,77,78,79], six in the 2000s [30,80,81,82,83,84], one in the 1990s [85], and one in the 1980s [86]. One guideline had no publication date [87].

### 3.1. Guideline Development Methodology

A total of 34/58 guidelines did not provide any explanation of the methods employed for guideline development. Of the guidelines that did describe the methods, 20 provided a grading of the evidence. To assess the certainty of evidence and strength of recommendations, the most employed method was the GRADE approach, used in eight guidelines [50,53,60,62,68,71,72,74]. Other methods included the Oxford Centre for Evidence Based Medicine (*n* = 2) [63,76], Scottish Intercollegiate Guidelines Network (*n* = 1) [33], National Health and Medical Research Council (*n* = 1) [38], National Comprehensive Cancer Network (*n* = 1) [66], Infectious Disease Society of America—US public health service grading system (*n* = 1) [49], Canadian Task Force on Preventive Health Care to grade recommendations (*n* = 1) [44], British Committee for Standards in Haematology (*n* = 1) [45], and Minds2014 (*n* = 1) [59]. Three guidelines did not specify which grading system was used [80,83,84].

### 3.2. Types of Iron Deficiency

A total of 24/33 general and 4/10 pregnancy-specific guidelines focused only on IDA [30,32,33,34,36,37,38,39,40,42,43,46,52,53,54,55,57,58,70,71,73,76,77,80,81,85,86,87], while 9 general and 6 pregnancy-specific guidelines included information on both IDA and non-anemic iron deficiency (NAID) [31,35,41,44,45,48,51,64,65,67,68,72,74,75,78].

### 3.3. Biomarkers

#### 3.3.1. Serum Ferritin and Other Iron Biomarkers

**Figure 3:**
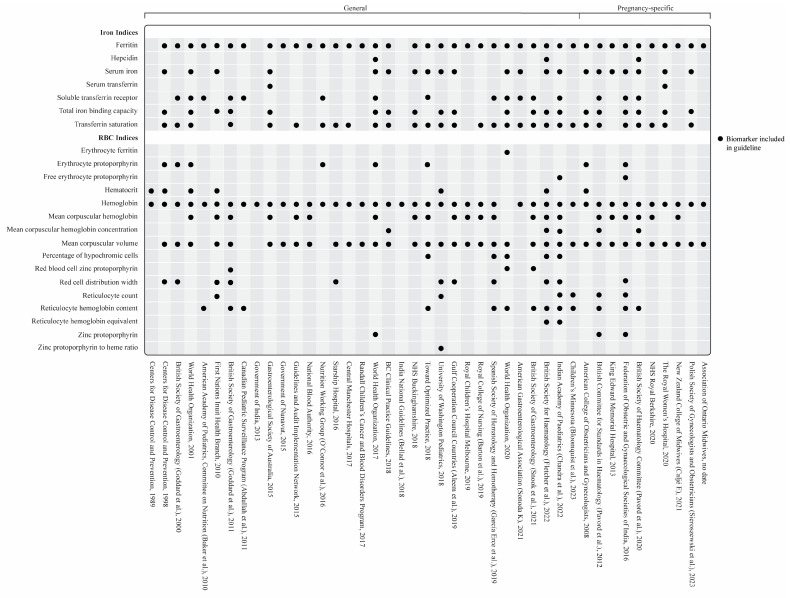
Recommended biomarkers for the diagnosis of NAID/IDA in the included general and pregnancy-specific guidelines [30,31,32,33,34,35,36,37,38,39,40,41,42,43,44,45,46,48,51,52,53,54,55,57,58,64,65,67,68,70,71,72,73,74,75,76,77,78,80,81,85,86,87].

**Figure 4:**
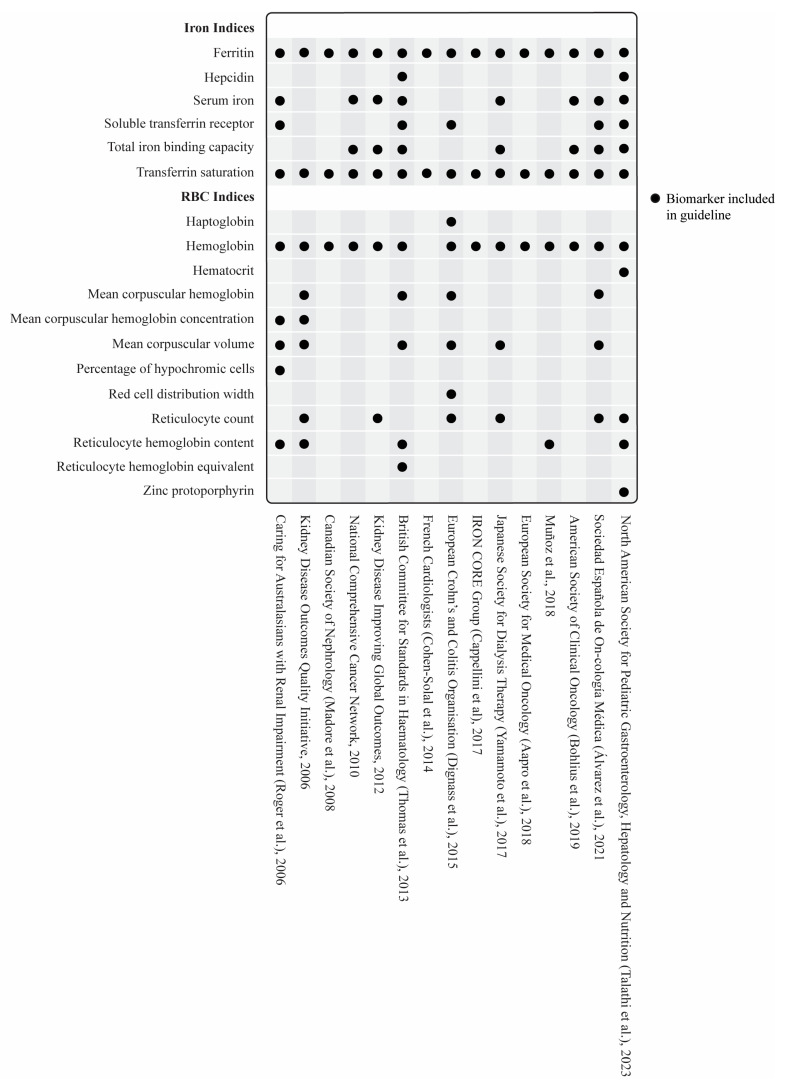
Recommended biomarkers for the diagnosis of NAID/IDA in the included disease-specific guidelines [47,49,50,56,59,60,61,62,63,66,69,79,82,83,84].

**Figure 5:**
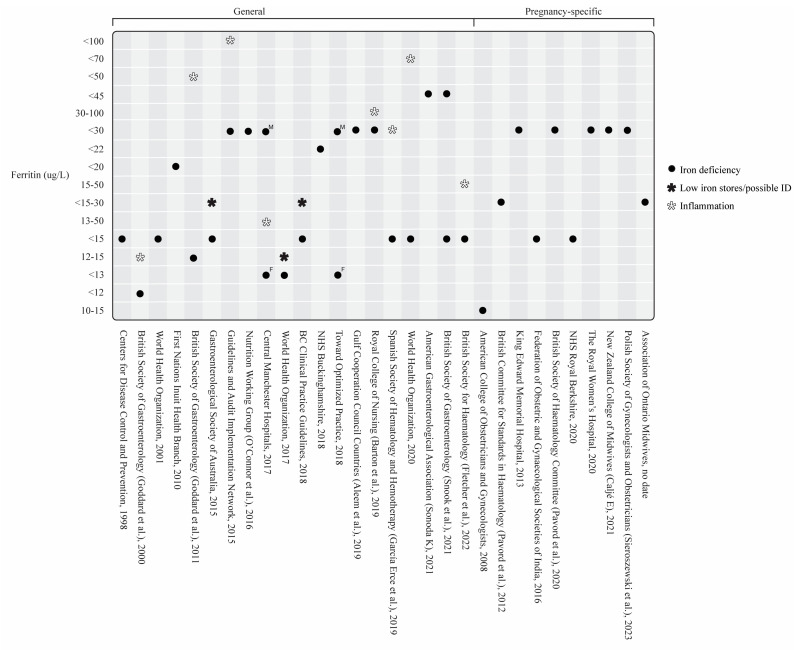
Serum ferritin cutoff values of the included general and pregnancy-specific guidelines. F: female; M: male [30,31,33,34,35,39,40,42,43,44,45,46,51,53,54,55,57,67,68,70,71,72,74,75,77,78,80,81,85,87].

**Figure 6:**
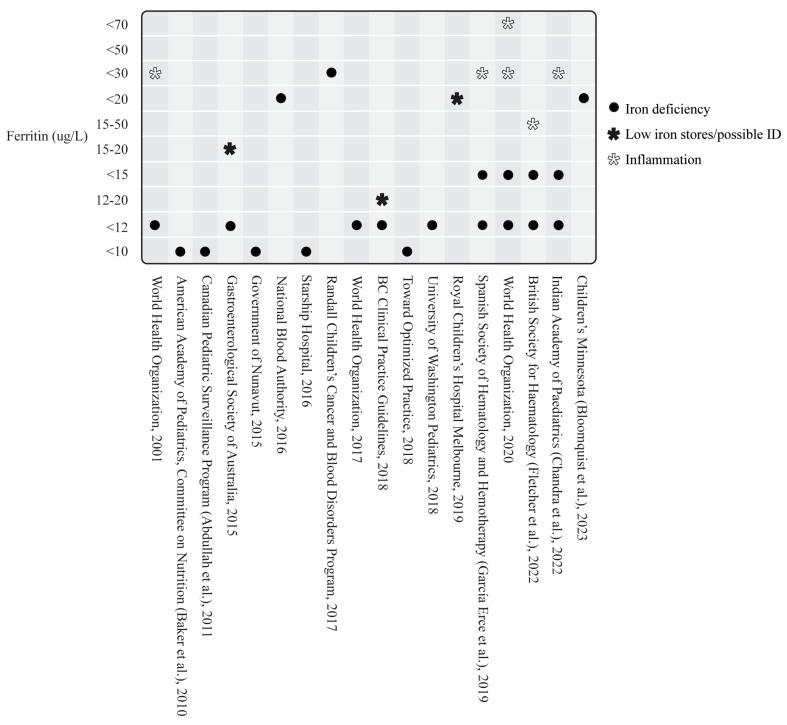
Pediatric-specific serum ferritin cutoff values of the included general ID guidelines [30,31,35,37,38,40,41,42,43,48,52,58,64,65,72,73,74,76].

A total of 54/58 guidelines recommended measuring SF and provided cutoff values [30,31,33,34,35,37,38,39,40,41,42,43,44,45,46,47,48,49,50,51,52,53,54,55,56,57,58,59,60,61,63,64,65,66,67,68,69,70,71,72,73,74,75,76,77,78,79,80,81,82,83,84,85,87]. Three guidelines did not include SF [32,36,86], and one included SF but did not include cutoff values [62]. Besides SF, the most commonly suggested iron-specific markers were transferrin saturation (*n* = 45), serum iron (*n* = 29), total iron binding capacity (*n* = 27), and soluble transferrin receptor (*n* = 21) (Figure 3 and Figure 4). Figure 5 and Figure 6 outline the SF cutoff values recommended by the included guidelines.

##### General Iron Deficiency Guidelines

SF cutoff values ranged from 10 ug/L to 100 ug/L in general ID guidelines. Two general ID guidelines had differing SF cutoff values for females and males, whereby females had lower SF cutoff values than males (<13 ug/L females; <30 ug/L males) [42,54]. Eighteen general ID guidelines had child-specific SF cutoff values shown in Figure 5 [30,31,35,37,38,40,41,42,43,48,52,58,64,65,72,73,74,76].

##### Iron Deficiency in Pregnancy

SF cutoff values ranged from <10 ug/L to <30 ug/L. Two guidelines had SF cutoff values of <15 ug/L but noted that treatment should be initiated when SF <30 ug/L [53,77]. One guideline recommended that, in non-anemic pregnant females with more than 16 weeks gestation, iron supplementation be commenced when SF <60 ug/L [78].

##### Iron Deficiency in Disease-Specific States

The most common condition was CKD (*n* = 5) [50,59,82,83,84] followed by cancer (*n* = 4) [49,62,66,69], inflammatory bowel disease (IBD) (*n* = 1) [63], intestinal rehabilitation (*n* = 1) [79], and heart failure (*n* = 1) [56]. One guideline included three distinct diseases (congestive heart failure (CHF), CKD, and IBD) [61]. One guideline was designed for use in post-operative patients [47]. In addition, one guideline was specifically looking at functional iron deficiency (FID), with an emphasis on patients with CKD [60].

SF cutoff values ranged from <12 ug/L to <800 ug/L. Higher cutoff values for specific subpopulations were suggested in eight disease-specific guidelines; for individuals with inflammation/anemia of chronic disease (*n* = 2) [63,69], on hemodialysis (*n* = 2) [60,84], those receiving an erythropoiesis stimulating agent (ESA) (*n* = 3) [50,59,83], and those on hemodialysis and receiving an ESA (*n* = 1) [82]. Two guidelines included FID [60,66], for which the following SF cutoff values were suggested: <100 ug/L (non-hemodialysis patients) and <200 ug/L (hemodialysis patients) [60], <800 ug/L [66]. One disease-specific ID guideline did not provide a SF cutoff value, though SF measurement was still suggested [62].

#### 3.3.2. Hematologic Biomarkers

Hemoglobin measurement was recommended in 56 ID guidelines. Of these 56, two guidelines used hemoglobin [32,36], and one used hemoglobin and hematocrit [86] as the only recommended biomarker(s) for detecting ID/IDA. One guideline did not explicitly state that hemoglobin should be measured in the context of diagnosing ID in heart failure [56]. Similarly, the WHO guideline on the use of ferritin did not include hemoglobin, though this guideline was focused on SF rather than ID/IDA as a broader concept [74]. A detailed overview of all hematologic biomarkers is shown in Figure 3 and Figure 4.

#### 3.3.3. Biomarkers of Inflammation

A total of 25/58 ID guidelines recommended the concomitant measurement of CRP with SF [31,37,38,39,43,45,48,49,50,51,54,55,57,58,61,63,64,69,70,71,72,73,74,75,79]. Of these, two guidelines from the WHO recommended both CRP and α−1 acid glycoprotein (AGP) [43,74]. An additional 23 guidelines acknowledged the role of SF as an acute phase reactant that can increase in the presence of inflammation and/or infection, though measurement of CRP or another inflammatory marker was not explicitly stated [30,33,34,35,40,41,42,44,46,47,53,56,59,60,65,67,68,76,78,81,83,85,87].

### 3.4. Iron Deficiency, ADHD, and RLS

Three guidelines included ADHD (two general, one disease-specific) [35,42,63] and nine guidelines included RLS (four general; three pregnancy-specific, two disease-specific) [31,42,53,58,61,63,68,71,75] as signs/symptoms of ID or as associated conditions. Of these, one general and one disease-specific guideline included both ADHD and RLS [42,63]. Importantly, the BC Clinical Practice Guideline (Canada) included a SF cutoff value of ≤75 ug/L for iron supplementation in individuals with RLS [31].

## 4. Discussion

### 4.1. Iron Deficiency

ID is a paraphysiological state that precedes anemia and is associated with restlessness that can affect day- and night-time behaviors. Recent research suggests that ID, and more specifically CID, may be a precipitating factor in conditions that can present with overall restlessness, such as ADHD and RLS, which are two of the most frequently encountered neurobehavioral and neurologic conditions, with a prevalence of 7% and 4–14%, respectively [89,90]. We reviewed ID guidelines to determine the recommended iron biomarkers, their cutoff values, the role of inflammation when interpreting laboratory results, and whether these common disorders were incorporated.

In this scoping review, we identified 58 ID guidelines published across five decades and 11 countries, including 6 international guidelines. Included in this analysis were three guidelines from the WHO published in 2001, 2017, and 2020, respectively [30,43,74]. Our findings demonstrate that (1) SF is the primary biomarker for ID, though cutoff values vary between guidelines, (2) ADHD and RLS have seldom been incorporated as associated conditions or signs of ID, and (3) inflammation needs to be taken into consideration (e.g., by measuring CRP and completing a physical examination) when interpreting SF. The majority of guidelines in this review focused on IDA, while a smaller proportion included information on NAID with a preponderance to more recently published guidelines. This likely reflects the increasing knowledge that ID, even in the absence of anemia, can have adverse consequences on health [91].

### 4.2. Serum Ferritin

A total of 54/58 guidelines included in this review recommended SF as one of the primary biomarkers for diagnosing ID. However, cutoff values varied between guidelines and based on additional factors such as age or sex. Over the past two decades, the WHO guidelines have recommended SF as the primary marker of ID and utilized the same cutoff values during this time period [30,43,74]. However, there is clinical controversy suggesting that the cutoff values by the WHO may actually be too low, and proposals for higher cutoff values have been made. Based on data from the US National Health and Nutrition Examination Survey, utilizing information from 2569 children and 7498 non-pregnant women, the cross-sectional study by Mei et al. concluded that SF cutoff values of 20 ug/L for children and 25 ug/L for non-pregnant women would be more appropriate [92].

Due to the role of SF as an acute phase reactant, the utilization of additional biomarkers plays a key role in the identification of iron-depleted states. The two most recent WHO guidelines recommend screening for ID with SF and, when appropriate, the concomitant measurement of CRP and/or AGP [43,74]. While CRP is a well-recognized and widely available marker of inflammation, in our experience, AGP is less frequently used in clinical practice. In comparison to CRP, the rise in AGP is slower but remains elevated for a longer time period after the initial inflammatory event [93].

### 4.3. ID-Associated Conditions

In two recent scoping reviews investigating the role of ID in neurodevelopmental disorders and in sleep, respectively, 22 out of 30 studies demonstrated an association of ID and ADHD [7], and 24 out of 47 studies showed a positive association between ID and RLS [10]. The inconsistency in the results of the studies included in these scoping reviews may be explained by the heterogeneity of methodologies and missing the role of inflammation as a factor affecting iron homeostasis, indicating the need for a harmonization of the approach to ID in these populations. The association between ID and conditions presenting with restlessness is hypothesized to be due, in part, to the role of brain iron.

In the current scoping review, nine studies mentioned RLS and only three mentioned ADHD as being associated with ID. These results are unsurprising given that studies implicating ID, and more specifically CID, are relatively recent, but this highlights the knowledge gaps that exist in current clinical practice. While there is limited evidence to demonstrate the effect of iron supplementation on brain iron levels directly, iron supplementation has been found to reduce symptom severity in both ADHD and RLS [7,10,94,95].

### 4.4. Brain Iron Imaging

The measurement of brain iron via in vivo brain magnetic resonance techniques is the most direct approach to CID. Recent advances in MRI have opened up new opportunities, allowing safe and non-invasive brain iron level estimates in vivo [96]. Currently, two main methods are applied for measuring brain iron: (a) methods based on transverse relaxation times; and (b) methods based on phase information to produce susceptibility weighted imaging (SWI) and quantitative susceptibility maps (QSMs). Iron, especially when it is stored in ferritin or hemosiderin, induces local magnetic field inhomogeneities and increases relaxation rates. These rates can be measured by using a multi-echo acquisition, then performing voxelwise fitting to an exponential model [97]. SWI is a weighted MRI technique that enhances image contrast by using the susceptibility differences between tissues [98].

In pediatric patients, the early diagnosis of neurodegeneration with brain iron accumulation has been shown with SWI, and SWI has been integrated successfully into fetal MRI studies [99,100]. QSMs provide quantitative measures of magnetic susceptibility and are based on paramagnetic effects from iron-containing proteins that increase bulk tissue magnetic susceptibility. QSMs are calculated through a multi-step process that ultimately converts phase information acquired via a weighted sequence into magnetic susceptibility values [101]. QSMs have also been used to describe adult disorders that are associated with iron overload, such as neurodegenerative disorders and multiple sclerosis [102] and may support phenotyping studies for describing the role of iron homeostasis. While few imaging studies exist for ADHD, the results so far demonstrate lower brain iron levels, and the systematic review by Degremont et al. concluded that brain iron, rather than serum iron levels, tend to be lower in children with ADHD [103]. While most brain imaging studies in individuals with RLS have shown lower brain iron levels, a recent meta-analysis of 72 patients with RLS showed the normal and increased iron content of subcortical brain areas, showing the complexity of capturing affected iron levels in the brain [19]. One of the limitations of both transverse relaxation and phase-based techniques is the fact that they are not specific to iron. All matter has a diamagnetic or paramagnetic component to it and ultimately contributes to shifting the phase or transverse relaxation, with iron being one of the strongest contributors [104]. Thus, while an increase in paramagnetism would suggest an increase in iron, it could also be from a loss of diamagnetic tissue or an increase in some other paramagnetic component, for example. Furthermore, other aspects such as the iron oxidation state could further bias the quantification of iron with MRI [105]. Ultimately, the authors of the meta-analysis conclude that brain iron mobilization or homeostasis is impaired in RLS, possibly through a reduction in the functional availability of iron or as a function of a decreased prevalence of transferrin receptors [87,89]. These results raise questions, not only about the methodologies of imaging studies but also about how to capture pathophysiologic mechanisms that modulate iron homeostasis. Such contradictions have inspired the title of our research endeavor, the ‘Iron Conundrum’. To fully understand the role of iron in RLS, extensive post-mortem studies in large cohorts will be necessary, regardless of whether new methods to evaluate brain iron metabolism are developed in the future.

### 4.5. Possible Pathophysiological Mechanisms of Central Iron Deficiency

Animal studies utilizing dietary modifications provide strong evidence for the brain iron mobilization concept and demonstrate causal links between ID and alterations in dopamine transmission at multiple levels that are consistent with the hypothesis of a CID-induced dysregulation of dopamine receptors in RLS (Figure 1) [2]. The proposed mechanism is the impaired mobilization of iron in the brain, leading to a functional ID [19]. Connor et al. demonstrated, in a small sample of four patients with early-onset RLS, that transferrin receptor expression was decreased [106]. Further supporting this hypothesis of impacted iron homeostasis and CID are studies that have shown significantly increased hepcidin levels in individuals with ADHD and RLS compared to controls [107,108,109,110]. Hepcidin is the primary regulator of iron homeostasis and works to block the efflux of cellular iron. It is highly expressed during states of normal iron homeostasis and suppressed during states of systemic ID. Inflammatory states are associated with increased levels of hepcidin and, thus, decreased mobilization of iron to the plasma and absorption of iron from the gastrointestinal tract [1,110]. Altogether, these factors result in the insufficient incorporation of iron into erythroid precursors in the face of seemingly normal peripheral iron stores [60]. Importantly, animal studies have also implicated hepcidin as a key player of iron homeostasis in the brain [111]. Due to functional limitations on how to assess iron function in the spinal cord, the majority of clinical and animal studies have focused on ID-induced alterations in the brain, thereby giving rise to the term “brain iron deficiency” [2]. However, a more recent study indicates that the effects of low iron levels in a non-anemic state can also be detected by an increased expression of the transferrin receptor in the spinal cord and an augmented sensorimotor excitability [3], thus warranting the use of the wider term “central iron dysfunction”, rather than only referring to the brain.

### 4.6. Clinical Perspectives and Future Research

As stated in this review, most recommendations, including the WHO guidelines, have not incorporated conditions presenting with restlessness, such as ADHD and RLS, which opens many avenues for therapeutic interventions, a critical analysis of existing guidelines, and an evaluation of the suggested changes with future clinical and experimental research. The reality is that while the need to be aware of iron homeostasis and/or CID concepts has been acknowledged among clinicians and researchers working in the field of sleep medicine, these concepts have not yet been established in daytime-focused conditions such as ADHD, despite being a 24-hour disorder. Therefore, the interpretation of iron status in individuals with conditions such as ADHD and RLS still poses a unique challenge for clinicians.

The explorative neurophysiological characterization of ADHD sleep phenotypes and the clinical approach to RLS from a patient perspective have been important first steps of clinical phenotyping [112,113,114,115]. Now, we need a standardization of semiology to capture the same clinical phenotypes. Given that conditions presenting with restlessness are thought to be primarily due to impaired iron homeostasis and/or central iron dysfunction, peripheral iron levels (e.g., measured by SF) may not be helpful at guiding clinical decision making using existing ID cutoff values, particularly if we do not proactively investigate inflammation. The well-established RLS treatment guidelines that use SF cutoff values of 75 ug/L and 50 ug/L for adults (including pregnancy) and children, respectively [115,116,117], are in contrast to the majority of general ID guidelines that use much lower cutoff values for the diagnosis of absolute ID (Figure 5 and Figure 6). At this stage, we suggest a consensus approach for harmonized ID screening with a focus on inflammatory states and, second, the utilization of the RLS-specific SF cutoff values, as currently, ADHD-specific cutoff values do not exist. At present, universal screening for ID/IDA is not recommended in otherwise healthy children [118]. However, a recent study by Carsley et al. suggest that screening for ID in young children would be cost-effective, citing the effects of ID on development and cognitive function [119]. As sleep disturbances, particularly those presenting with restlessness (e.g., RLS, PLMS, RSD), often pose a challenge in clinical practice, we are in support of such a screening concept in children and adolescents with neurodevelopmental disorders and/or mental health conditions [7,10,120,121]. Whether this should be broadened to a universal screening concept in otherwise healthy children is a topic of further discussion. As mentioned above, cutoff values in the context of sleep disturbances and neurodevelopmental and/or mental health conditions have to be higher than for the diagnosis of absolute ID. The integration of ADHD and RLS into general ID guidelines will therefore catalyze this endeavor.

Finally, there appear to be several avenues for CID research through brain imaging. One such method involves decomposing the QSM signal into its diamagnetic and paramagnetic components. Iron and deoxygenated blood are highly paramagnetic and result in positive values in QSM, and diamagnetic tissue such as myelin result in negative values. When a voxel contains both diamagnetic and paramagnetic tissue, these values will subtract from each other, masking both kinds of tissue. Using a method such as DECOMPOSE-QSM or APART-QSM would allow for separate diamagnetic and paramagnetic maps, improving the sensitivity and specificity for iron [122,123]. Another avenue is the fact that, previously, most clinically acquired SWI scans did not save the unfiltered phase images. QSM requires an unfiltered phase image in order to be calculated. Recent studies, using deep learning algorithms, have been trained to recover the unfiltered phase information from the filtered phase data [19,124]. Thus, many previous studies that could not be used to calculate QSM can now be carried out retrospectively, resulting in a potentially vast amount of ID data to be analyzed. In summary, the inclusion of QSM in MRI studies of patients with restlessness and a review of iron status might be the next avenue to explore in future studies.

## 5. Limitations

Despite conducting a scoping literature review, most guidelines were identified by searching the gray literature, which consisted of Google searches, searching websites of international, national, and regional health organizations, and utilizing the Trip Medical Database to identify guidelines that met our inclusion criteria. Thus, it is very likely that additional guidelines exist and were not included in this review, which could result in changes to our discussion and conclusions. Similarly, guidelines not published in English were excluded. Due to the fact that many of the included guidelines were found on websites, there is also a possibility that guidelines may be removed and/or updated, with the original guideline no longer available. In addition, our results show that the methodology for guideline development varied, with over half of the included guidelines not providing an explanation of their methods; this lack of transparency may have implications for guideline rigor and accuracy. However, this topic is outside the scope of this review and, therefore, was not analyzed in more detail.

One further limitation is the lack of accurate semiology. After in-depth discussions, the authors decided to use the term ‘restlessness’ as an umbrella term. However, as restlessness alters tone and posture, both of which are indicative of vigilance alterations, we may need to revisit clinical phenotyping terminology using the descriptions of visible motor and vigilance states for sleep and wake behaviors. This would then allow for capturing a broader spectrum of movement patterns; on the one side, hyperkinesia, presenting with a hypermotor and hypo- or hypervigilant state [112,125], and on the other hand, with hypokinesia, presenting with hypo- or hypervigilance [126], but also neurodiverse patients with restrictive hypokinesia/hypomotor behaviors [127]. Given the lack of consensus on phenotyping terminology, our provocative simplification might also be considered as a strength, as it starts the necessary semiology discourse.

## 6. Conclusions

The existing ID guidelines analyzed in this scoping review highlight the gaps in clinical knowledge, particularly when it comes to the concept of CID and restlessness. The majority of guidelines do not include ADHD or RLS. Further, the heterogeneity of biomarkers and SF cutoff values raises questions about the application of current ID guidelines in clinical practice. Given this, the next steps will be to (a) achieve consensus on how to investigate and interpret iron status as part of a homeostatic system (affected by age, sex, pregnancy, inflammation, and comorbidities), (b) develop screening criteria for conducting iron studies, (c) incorporate the concept of iron homeostasis and CID into clinical practice guidelines, and (d) conduct further brain imaging studies to better elucidate the role of iron in the pathophysiology of conditions presenting with restlessness in sleep and wake states.

## Figures and Tables

**Table 2:** Search strategy used in CINAHL, Embase, and Medline.

Database		
CINAHL	(MH “Anemia, Iron Deficiency”) OR (TI “iron deficiency anemia” OR AB “iron deficiency anemia”) OR (TI “iron deficiency” OR AB “iron deficiency”)	(MH “Practice Guidelines”) OR (TI guideline or AB guideline)
Embase	(iron deficiency/) OR (iron deficiency anemia/) OR (iron deficiency anemia. tw, kw.) OR (iron deficiency. tw, kw.)	(practice guideline/) OR (guideline. tw, kw.)
Medline	(Anemia, Iron Deficiency/) OR (iron deficiency. tw, kf.) OR (iron deficiency anemia. tw, kf.)	(Practice Guideline/OR Guideline/) OR (Guideline. tw, kf.)

## Appendix A

**Table A1:** Preferred Reporting Items for Systematic reviews and Meta-Analyses extension for Scoping Reviews (PRISMA-ScR) Checklist [128].

Section	Item	PRISMA-ScR Checklist Item	Reported on Page #
**Title**			
Title	1	Identify the report as a scoping review.	1
**Abstract**			
Structured summary	2	Provide a structured summary that includes (as applicable): background, objectives, eligibility criteria, sources of evidence, charting methods, results, and conclusions that relate to the review questions and objectives.	1
**Introduction**			
Rationale	3	Describe the rationale for the review in the context of what is already known. Explain why the review questions/objectives lend themselves to a scoping review approach.	2,3,5
Objectives	4	Provide an explicit statement of the questions and objectives being addressed with reference to their key elements (e.g., population or participants, concepts, and context) or other relevant key elements used to conceptualize the review questions and/or objectives.	5
**Methods**			
Protocol and registration	5	Indicate whether a review protocol exists; state if and where it can be accessed (e.g., a Web address); and if available, provide registration information, including the registration number.	5
Eligibility criteria	6	Specify characteristics of the sources of evidence used as eligibility criteria (e.g., years considered, language, and publication status), and provide a rationale.	6
Information sources *	7	Describe all information sources in the search (e.g., databases with dates of coverage and contact with authors to identify additional sources), as well as the date the most recent search was executed.	5,6
Search	8	Present the full electronic search strategy for at least 1 database, including any limits used, such that it could be repeated.	Table 2
Selection of sources of evidence †	9	State the process for selecting sources of evidence (i.e., screening and eligibility) included in the scoping review.	5,6
Data charting process ‡	10	Describe the methods of charting data from the included sources of evidence (e.g., calibrated forms or forms that have been tested by the team before their use, and whether data charting was carried out independently or in duplicate) and any processes for obtaining and confirming data from investigators.	6
Data items	11	List and define all variables for which data were sought and any assumptions and simplifications made.	6,7
Critical appraisal of individual sources of evidence §	12	If performed, provide a rationale for conducting a critical appraisal of included sources of evidence; describe the methods used and how this information was used in any data synthesis (if appropriate).	N/A
Synthesis of results	13	Describe the methods of handling and summarizing the data that were charted.	6,7
**Results**			
Selection of sources of evidence	14	Give numbers of sources of evidence screened, assessed for eligibility, and included in the review, with reasons for exclusions at each stage, ideally using a flow diagram.	7, Figure 2
Characteristics of sources of evidence	15	For each source of evidence, present characteristics for which data were charted and provide the citations.	7,8,9,11,12,13, Table A2, Table A3 and Table A4
Critical appraisal within sources of evidence	16	If performed, present data on critical appraisal of included sources of evidence (see item 12).	N/A
Results of individual sources of evidence	17	For each included source of evidence, present the relevant data that were charted that relate to the review questions and objectives.	7,8,9,11,12,13, Table A2, Table A3 and Table A4, Figure 3, Figure 4, Figure 5 and Figure 6
Synthesis of results	18	Summarize and/or present the charting results as they relate to the review questions and objectives.	7,8,9,11,12,13,
**Discussion**			
Summary of evidence	19	Summarize the main results (including an overview of concepts, themes, and types of evidence available), link to the review questions and objectives, and consider the relevance to key groups.	13,14,15,16,17
Limitations	20	Discuss the limitations of the scoping review process.	17
Conclusions	21	Provide a general interpretation of the results with respect to the review questions and objectives, as well as potential implications and/or next steps.	17
**Funding**			
Funding	22	Describe sources of funding for the included sources of evidence, as well as sources of funding for the scoping review. Describe the role of the funders of the scoping review.	17

JBI = Joanna Briggs Institute; PRISMA-ScR = Preferred Reporting Items for Systematic reviews and Meta-Analyses extension for Scoping Reviews. * Where sources of evidence (see second footnote) are compiled from, such as bibliographic databases, social media platforms, and Web sites. † A more inclusive/heterogeneous term used to account for the different types of evidence or data sources (e.g., quantitative and/or qualitative research, expert opinion, and policy documents) that may be eligible in a scoping review as opposed to only studies. This is not to be confused with information sources (see first footnote). ‡ The frameworks by Arksey and O’Malley and Levac and colleagues and the JBI guidance refer to the process of data extraction in a scoping review as data charting. § The process of systematically examining research evidence to assess its validity, results, and relevance before using it to inform a decision. This term is used for items 12 and 19 instead of “risk of bias” (which is more applicable to systematic reviews of interventions) to include and acknowledge the various sources of evidence that may be used in a scoping review (e.g., quantitative and/or qualitative research, expert opinion, and policy document).

**Table A2:** Overview of included general ID guidelines. ✓: mentioned; ⨯: not mentioned; * Does not explicitly state CRP, but discusses ferritin as an acute phase reactant; ADHD: attention-deficit/hyperactivity disorder; CHr: reticulocyte hemoglobin content; CRP: C reactive protein; EP: erythrocyte protoporphyrin; F: females; Hb: hemoglobin; Hct: hematocrit; IDA: iron deficiency anemia; Intl: international; M: males; MCH: mean corpuscular hemoglobin; MCV: mean corpuscular volume; MCHC: mean corpuscular hemoglobin concentration; NAID: non-anemic iron deficiency; NZ: New Zealand; PV: plasma viscosity; Retic. count: reticulocyte count; rbZP: red blood cell zinc protoporphyrin; Ret-HE: reticulocyte hemoglobin equivalent; RDW: red cell distribution width; RLS: restless legs syndrome; SES: socioeconomic status; SI: serum iron; sTfR: soluble transferrin receptor; TIBC: total iron binding capacity; TSAT: transferrin saturation; UK: United Kingdom; USA: United States of America; ZPP: zinc protoporphyrin; ZPPH: zinc protoporphyrin to heme ratio; %HRC: percentage hypochromic red cells.

Guideline	Country/Region	Type of ID	Population	C R P	A D H D	R L S	Serum Ferritin (ug/L)	Additional Biomarkers	Risk Factors for ID/IDA
Centers for Disease Control and Prevention, 1989 [86]	USA	IDA	Children and females	⨯	⨯	⨯	N/A	-Hb -Hct	-Infants/ children -Females of child-bearing age -Pregnancy
Centers for Disease Control and Prevention, 1998 [85]	USA	IDA	Children and adults	⨯ *	⨯	⨯	>6 months: ≤15	-EP -Hb -Hct -MCV -RDW -SI -TIBC -TSAT	-Infants/ children -Females of child-bearing age -Infant diet -Diet -Infection/ inflammation/ comorbidities -Pregnancy -Prematurity/ LBW -Medications -Low SES -Race/ ethnicity
British Society of Gastroenterology (Goddard et al.), 2000 [81]	UK	IDA	Adults	⨯ *	⨯	⨯	<12 12–15 in the presence of inflammation	-EP -Hb -MCV -RDW -sTfR -TSAT	-Menstruation -Infection/ inflammation/ comorbidities
World Health Organization, 2001 [30]	Intl	IDA	Children and adults	⨯ *	⨯	⨯	<5 years: <12 <30 in the presence of infection >5 years: <15	-EP -Hb -Hct -MCH -MCV -SI -sTfR -TIBC -TSAT	-Menstruation -Pregnancy -Infection/ inflammation/ comorbidities -Diet -Low SES -Infant diet
American Academy of Pediatrics, Committee on Nutrition (Baker et al.), 2010 [64]	USA	IDA NAID	Infants and children 0–3 years	✓	⨯	⨯	0–3 years: <10	-CHr -Hb -STfR	-Prematurity/ LBW -Infant diet -Lead exposure -Poor growth -Low SES
First Nations and Inuit Health Branch, 2010 [34]	Canada	IDA	Adults	⨯ *	⨯	⨯	<20	-Hb -Hct -MCH -MCV -RDW -Retic. count -SI -TIBC	-Females of child- bearing age -Infants/ children, -Adolescents -Advanced age -Infection/ inflammation/ comorbidities -Race/ ethnicity -Menstruation
British Society of Gastroenterology (Goddard et al.), 2011 [33]	UK	IDA	Adults	⨯ *	⨯	⨯	<12–15 <50 in the presence of inflammation	-CHr -Hb -MCH -MCV -rbZP -RDW -sTfR -TIBC -TSAT	-Infection/ inflammation/ comorbidities
Canadian Pediatric Surveillance Program (Abdullah et al.), 2011 [48]	Canada	IDA NAID	Children	✓	⨯	⨯	<10	-CHr -Hb -sTfR	-Low SES -Race/ ethnicity -Prematurity/ LBW -Infant diet -Infection/ inflammation/ comorbidities -Non-attendance to daycare -Overweight/obesity
Government of India, 2013 [36]	India	IDA	Adults and children	⨯	⨯	⨯	N/A	-Hb	-Females of childbearing age -Infants/ children -Infection/ inflammation/ comorbidities -Diet -Infant diet
Gastroenterol-gical Society of Australia, 2015 [35]	Australia	IDA NAID	Adults and children	⨯ *	✓	⨯	Children: <12 diagnostic <15–20 low iron stores Adults: <15 diagnostic <15–30 low iron stores	-Hb -MCH -MCV -SI -TIBC -Serum transferrin -TSAT	-Pregnancy -Females of child-bearing age -Infants/ children -Infection/ inflammation/ comorbidities -Diet -Advanced age -Race/ ethnicity -Elite athletes -Overweight/obesity
Government of Nunavut, 2015 [37]	Canada	IDA	Infants and children 6 mo–3 years	✓	⨯	⨯	<10	-Hb -MCV	-Low SES -Infant diet
Guidelines and Audit Implementati- on Network, 2015 [55]	UK	IDA	Adults	✓	⨯	⨯	CRP < 30 mg/L: <30 CRP > 30 mg/L: <100	-Hb -MCH -MCV -TSAT	-Infection/ inflammation/ comorbidities -Pregnancy -Menstruation
National Blood Authority, 2016 [38]	Australia	IDA	Children	✓	⨯	⨯	<20 High risk populations/chronic disease: <50	-Hb -MCH -MCV	-Race/ ethnicity -Maternal ID -Prematurity/ LBW -Infant diet -Infection/ inflammation/ comorbidities
Nutrition Working Group (O’Connor et al.), 2016 [44]	Canada	IDA NAID	Females (adolescence to menopause)	⨯ *	⨯	⨯	<30	-EP -Hb -sTfR -TSAT	-Diet -Low SES -Race/ ethnicity -Menstruation -Pregnancy -Overweight/ obesity
Starship Hospital, 2016 [41]	NZ	IDA NAID	Children age 1–5 years	⨯ *	⨯	⨯	<10	-Hb -TSAT -MCV -RDW	-Infants/ children -Race/ ethnicity -Prematurity/ LBW -Maternal ID -Overweight/ obesity -Adolescence -Diet -Infant diet -Menstruation
Central Manchester Hospitals, 2017 [54]	UK	IDA	Non- pregnant adults	✓	⨯	⨯	<13 (F) <30 (M) Elevated ESR/PV: 13–50	-Hb -MCV -TSAT	-Infection/ inflammation/ comorbidities
Randall Children’s Cancer and Blood Disorders Program, 2017 [52]	USA	IDA	Children	⨯	⨯	⨯	<30	-Hb -MCV	-Infection/ inflammation/ comorbidities
World Health Organization, 2017 [43]	Intl	IDA	Children and adults	✓	⨯	⨯	<5 years: <12 >5 years: <15	-EP -Hb -Hepcidin -MCH -MCV -SI -sTfR -TIBC -TSAT -ZPP	-Infants/ children -Menstruation -Pregnancy -Advanced age
BC Clinical Practice Guidelines, 2018 [31]	Canada	IDA NAID	Adults and children	✓	⨯	✓	Children: <12 12–20 possible ID Adults: <15 15–30 probable ID	-Hb -MCHC -MCV -SI -TIBC -TSAT	-Pregnancy -Infants/ children - Low SES -Ethnicity/race -Diet -Infection/ inflammation/ comorbidities -Advanced age -Menstruation
India National Guidelines (Bellad et al.), 2018 [32]	India	IDA	Children aged 6 month–14 years, non-pregnant females age > 15, pregnant females	⨯	⨯	⨯	N/A	-Hb	-Pregnancy, -Infants/ children -Adolescents -Infection/ inflammation/ comorbidities
NHS Buckinghams-hire, 2018 [57]	UK	IDA	Adults	✓	⨯	⨯	<22	-Hb -MCH -MCV -SI -TIBC -TSAT	-Menstruation -Infection inflammation/ comorbidities
Toward Optimized Practice Alberta, 2018 [42]	Canada	IDA	Children (≥5 years) and adults	⨯ *	✓	✓	<12 years: <10 >12 years: <13 F <30 M	-CHr -EP -Hb -%HRC -MCH -MCV -SI -sTfR -TSAT	-Infants/ children -Adolescence -Menstruation -Pregnancy -Low SES -Diet -Advanced age -Infection/ inflammation/ comorbidities
University of Washington Pediatrics, 2018 [58]	USA	IDA	Children	✓	⨯	✓	<5 years: <12 ≥5 years: <15	-Hb -Hct -MCV -RDW -Retic. count -SI -TIBC -TSAT -ZPPH	-Prematurity/ LBW -Poor growth/FTT -Infant diet -Diet -Lead exposure -Infection/ inflammation/ Comorbidities -Overweight/ obesity -Menstruation
Gulf Cooperation Council Countries (Aleem et al.), 2019 [46]	Gulf Coop Countries	IDA	Adults and children	⨯ *	⨯	⨯	<30 (ID should still be considered in high-risk patients with SF > 30)	-Hb -MCH -MCV -RDW -SI -TIBC -TSAT	-Pregnancy -Infant feeding -Prematurity/ LBW -Overweight/ obesity
Royal Children’s Hospital Melbourne, 2019 [65]	Australia	IDA NAID	Children	⨯ *	⨯	⨯	<20	-Hb -MCH -MCV	-Maternal ID -Prematurity/ LBW -Pregnancy -Infant diet -Diet -Infection/ inflammation/ comorbidities -Menstruation -Extreme athletes
Royal College of Nursing (Barton et al.), 2019 [39]	UK	IDA	Adults	✓	⨯	⨯	<30 CRP > 5 mg/L: 30–100	-Hb -MCH -MCV -TSAT	-Diet -Infection/ inflammation/ comorbidities
Spanish Society of Hematology and Hemotherapy (García Erce et al.), 2019 [40]	Spain	IDA	Children and adults	⨯ *	⨯	⨯	≤ 5 years: <12 >5 years: <15 <30 in the presence of inflammation Athletes > 15 years: <30	-CHr -Hb -%HRC -MCH -MCV -RDW -sTfR -TSAT	-Pregnancy -Elite athletes -Infant diet -Adolescents
World Health Organization, 2020 [74]	Intl	IDA NAID	Children and adults	✓	⨯	⨯	<5 years: <12 <30 in the presence of infection/ inflammation 5–10 years, adolescents, adults, elderly, 1st trimester pregnancy: <15 <70 in the presence of infection/ inflammation	-CHr -Erythrocyte ferritin -%HRC -MCV -rbZP -SI -sTfR -TIBC -TSAT	N/A
American Gastroenterol-ogical Association (Sonoda K), 2021 [70]	USA	IDA	Adults	✓	⨯	⨯	<45 (with anemia) Ferritin threshold for ID without anemia is uncertain	-Hb -SI -sTfR -TSAT	-Females of child-bearing age -Infection/ inflammation/ comorbidities
British Society of Gastroenterol-ogy (Snook et al.), 2021 [71]	UK	IDA	Adults	✓	⨯	✓	<15 (highly specific for ID) <45 (respectable specificity for ID)	-CHr -Hb -MCH -MCV -rbZP -sTFR -TIBC -TSAT	-Menstruation -Infection/ inflammation/ comorbidities
British Society for Haematology (Fletcher et al.) 2022 [72]	UK	IDA NAID	Non-pregnant adults and children	✓	⨯	⨯	<5 years: <12 >5 years: <15 <15 or 15–50 in the presence of inflammation or raised CRP	-CHr -Hb -Hct -Hepcidin -%HRC -MCH -MCHC -MCV -RDW -Ret-HE -SI -TIBC -TSAT	-Menstruation -Advanced age
Indian Academy of Pediatrics (Chandra et al.), 2022 [76]	India	IDA	Children	⨯ *	⨯	⨯	<5 years: <12 >5 years: <15 <30 in the presence of infection	-CHr -FEP -Hb -%HRC -MCH -MCHC -MCV -RDW -Ret-HE -Retic. count -SI -sTFR -TIBC -TSAT	-Prematurity /LBW -Infants/ children -Adolescents
Children’s Minnesota (Bloomquist et al.), 2023 [73]	USA	IDA	Children ≤ 5 years	✓	⨯	⨯	<12 (goal > 20)	-CHr -Hb -TSAT -MCV -Retic. count	-Diet -Infection/ inflammation/ comorbidities

**Table A3:** Overview of included pregnancy-specific ID guidelines. ✓: mentioned; ⨯: not mentioned; * Does not explicitly state CRP, but discusses ferritin as an acute phase reactant; ADHD: attention-deficit/hyperactivity disorder; CHr: reticulocyte hemoglobin content; CRP: C reactive protein; EP: erythrocyte protoporphyrin; FEP: free erythrocyte protoporphyrin; Hb: hemoglobin; Hct: hematocrit; IDA: iron deficiency anemia; MCH: mean corpuscular hemoglobin; MCHC: mean corpuscular hemoglobin concentration; MCV: mean corpuscular volume; NAID: non-anemic iron deficiency; Retic. count: reticulocyte count; RDW: red cell distribution width; RLS: restless legs syndrome; SI: serum iron; sTFR: soluble transferrin receptor; TIBC: total iron binding capacity; TSAT: transferrin saturation; UK: United Kingdom. USA: United States of America; ZPP: zinc protoporphyrin; Chronic disease includes the following disease processes: infection, renal disease, gastrointestinal disease, malignancy, and cardiac disease.

Guideline	Country/ Region	Type of Iron Deficiency	C R P	A D H D	R L S	Serum Ferritin (ug/L)	Additional Biomarkers
American College of Obstetricians and Gynecologists, 2008 [80]	USA	IDA	⨯	⨯	⨯	<10–15	-EP -Hb -Hct -MCV -TIBC -TSAT -SI
British Committee for Standards in Haematology (Pavord et al.), 2012 [45]	UK	IDA NAID	✓	⨯	⨯	<15–30	-CHr -Hb -MCH -MCHC -MCV -Retic. count -SI -sTfR -TIBC -TSAT -ZPP
King Edward Memorial Hospital, 2013 [51]	Australia	IDA NAID	✓	⨯	⨯	<30	-Hb -MCH -MCV -SI
The Federation of Obstetric and Gynaecological Societies of India, 2016 [53]	India	IDA	⨯ *	⨯	✓	<15 (initiate treatment if <30)	-CHr -EP -FEP -Hb -MCH -MCV -RDW -Retic. count -SI -sTfR -TIBC -TSAT -ZPP
British Society of Hematology Committee (Pavord et al.), 2020 [68]	UK	IDA NAID	⨯ *	⨯	✓	<30	-CHr -Hb -Hepcidin -MCH -MCHC -MCV -TIBC -TSAT -SI -sTfR
NHS Royal Berkshire, 2020 [77]	UK	IDA	⨯	⨯	⨯	<15 (consider treatment if <30)	-Hb -MCH -MCV -TSAT
The Royal Women’s Hospital, 2020 [67]	Australia	IDA NAID	⨯ *	⨯	⨯	<30	-Hb -MCV -Serum transferrin -SI -TIBC -TSAT
New Zealand College of Midwives (Caljé E), 2021 [75]	New Zealand	IDA NAID	✓	⨯	✓	<30	-Hb -MCH -MCV
Polish Society of Gynecologists and Obstetricians (Sieroszewski et al.), 2023 [78]	Poland	IDA NAID	⨯ *	⨯	⨯	<30 (pre-latent) <12 (latent) Supplement when SF < 60 in pregnant females > 16 weeks of gestation	-Hb -MCV -SI -TIBC -TSAT
Association of Ontario Midwives, no date [87]	Canada	IDA	⨯ *	⨯	⨯	<15–30	-Hb -MCV

**Table A4:** Overview of included disease-specific ID guidelines. ✓: mentioned; ⨯: not mentioned; * Does not explicitly state CRP, but discusses ferritin as an acute phase reactant; ADHD: attention deficit hyperactivity disorder; CHF: congestive heart failure; CHr: reticulocyte hemoglobin content; CKD: chronic kidney disease; CRP: C reactive protein; Hb: hemoglobin; Hct: hematocrit; HD-CKD: hemodialysis-chronic kidney disease; IBD: inflammatory bowel disease; ID: iron deficiency; MCHC: mean corpuscular hemoglobin concentration; MCV: mean corpuscular volume; non-HD-CKD: non-hemodialysis chronic kidney disease; RDW: red cell distribution width; Ret-HE: reticulocyte hemoglobin equivalent; Retic. count: reticulocyte count; RLS: restless legs syndrome; sTfR: soluble transferrin receptor; TIBC: total iron binding capacity; TSAT: transferrin saturation; UK: United Kingdom; USA: United States of America; ZPP: zinc protoporphyrin.

Guideline	Country/Region	C R P	A D H D	R L S	Condition	Serum Ferritin	Additional Biomarkers
Caring for Australasians with Renal Impairment (Roger et al.), 2006 [83]	Australia	⨯ *	⨯	⨯	Kidney disease	<500 (commence IV iron in patients receiving ESA)	-CHr (research only) -Hb -% HRC -TSAT -MCHC -MCV -SI -sTfR
Kidney Disease Outcomes Quality Initiative, 2006 [84]	USA	⨯	⨯	⨯	Kidney disease	<200 (HD-CKD) <100 (non-HD-CKD)	-CHr -Hb -MCH -MCHC -MCV -Retic. count -TSAT
Canadian Society of Nephrology (Madore et al.), 2008 [82]	Canada	⨯	⨯	⨯	Kidney disease	<100 (non-dialysis) <200 (hemodialysis receiving ESA)	-Hb -TSAT
National Comprehensive Cancer Network, 2010 [66]	USA	⨯	⨯	⨯	Cancer	<30 (absolute ID) <800 (functional ID)	-Hb -SI -TIBC -TSAT
Kidney Disease Improving Global Outcomes, 2012 [50]	Intl	✓	⨯	⨯	Kidney disease	<500 (if being treated with ESA)	-Hb -Retic. count -SI -TIBC -TSAT
British Committee for Standards in Haematology (Thomas et al.), 2013 [60]	UK	⨯ *	⨯	⨯	Functional ID	<12 (absent iron stores) <100 (non- hemodialysis pt) <200 (hemodialysis pt)	-CHr -Hb -Hepcidin (research investigation only) -MCH -MCV -Ret-He -SI -sTfR -TIBC -TSAT
French Cardiologists (Cohen-Solal et al.), 2014 [56]	France	⨯ *	⨯	⨯	Heart failure	<100 Or 100–299 with TSAT < 20%	-TSAT
European Crohn’s and Colitis Organisation (Dignass et al.), 2015 [63]	Europe	✓	✓	✓	IBD	<30 (up to 100 in the presence of inflammation; >100 in anemia of chronic disease)	-Haptoglobin -Hb -MCH -MCV -RDW -Retic. count -sTfR -TSAT
IRON CORE Group (Cappellini et al.), 2017 [61]	Intl	✓	⨯	✓	Kidney disease CHF IBD	<100 (If SF 100–300, TSAT required for confirmation of ID)	-Hb -TSAT
Japanese Society for Dialysis Therapy (Yamamoto et al.), 2017 [59]	Japan	⨯ *	⨯	⨯	Kidney disease	<50 (if not treated with ESA) <100 (if being treated with ESA)	-Hb -MCV -Retic. count -SI -TIBC -TSAT
European Society for Medical Oncology (Aapro et al.), 2018 [49]	Europe	✓	⨯	⨯	Cancer	<100 (for patients receiving chemotherapy)	-Hb -TSAT
Muñoz et al., 2018 [47]	Intl	⨯ *	⨯	⨯	Post- operative	<100 Or 100–300 with TSAT < 20%	-CHr -Hb -TSAT
American Society of Clinical Oncology/American Society of Hematology (Bohlius et al.), 2019 [62]	USA	⨯	⨯	⨯	Cancer	No cutoff value (but recommend measuring ferritin)	-Hb -SI -TIBC -TSAT
Sociedad Española de Oncología Médica (Álvarez et al.), 2021 [69]	Spain	✓	⨯	⨯	Cancer	<30 30–100 (anemia of chronic disease with ID)	-Hb -MCH -MCV -Retic. count -SI -sTfR -TIBC -TSAT
North American Society for Pediatric Gastroenterology, Hepatology and Nutrition (Talathi et al.), 2023 [79]	North America	✓	⨯	⨯	Intestinal rehabilitation (e.g., in children with intestinal failure or short bowel syndrome)	<30 or 30–100 with TSAT < 15%	-CHr -Hb -Hct -Hepcidin (research setting) -Retic. count -SI -sTfR -TIBC -TSAT -ZPP
